# Biocompatible Lipid Polymer Cationic Nanoparticles for Antigen Presentation

**DOI:** 10.3390/polym13020185

**Published:** 2021-01-07

**Authors:** Yunys Pérez-Betancourt, Bianca de Carvalho Lins Fernandes Távora, Eliana L. Faquim-Mauro, Ana Maria Carmona-Ribeiro

**Affiliations:** 1 Biocolloids Laboratory, Departamento de Bioquímica, Instituto de Química, Universidade de São Paulo, Avenida Professor Lineu Prestes, 748 Butantan, São Paulo CEP 05508-000, Brazil; y.betancourt@usp.br; 2 Immunopathology Laboratory, Butantan Institute, Av. Vital Brasil, 1500 Butantan, São Paulo 05503-900, Brazil; bianca.tavora@butantan.gov.br (B.d.C.L.F.T.); eliana.faquim@butantan.gov.br (E.L.F.-M.)

**Keywords:** biocompatible polymer, cationic lipid, cationic polymer, ovalbumin, hybrid nanoparticles, humoral and cellular immune responses, protein delivery, vaccine adjuvants, cationic nanoparticles toxicity

## Abstract

Biocompatible lipid polymer nanoparticles (NPs) previously used as antimicrobial agents are explored here as immuno-adjuvants. Poly (methyl methacrylate) (PMMA)/dioctadecyldimethylammonium bromide (DODAB)/poly (diallyldimethylammonium chloride) (PDDA) nanoparticles (NPs) were prepared by emulsion polymerization of methyl methacrylate (MMA) in the presence of DODAB and PDDA, with azobisisobutyronitrile (AIBN) as the initiator. NPs characterization after dialysis by dynamic light-scattering yielded 225 ± 2 nm hydrodynamic diameter (Dz), 73 ± 1 mV zeta-potential (*ζ*), and 0.10 ± 0.01 polydispersity (P). Ovalbumin (OVA) adsorption reduced *ζ* to 45 ± 2 mV. Balb/c mice immunized with NPs/OVA produced enhanced OVA-specific IgG1 and IgG2a, exhibited moderate delayed type hypersensitivity reaction, and enhanced cytokines production (IL-4, IL-10, IL-2, IFN-γ) by cultured spleen cells. There was no cytotoxicity against cultured macrophages and fibroblasts. Advantages of the PMMA/DODAB/PDDA NPs were high biocompatibility, zeta-potential, colloidal stability, and antigen adsorption. Both humoral and cellular antigen-specific immune responses were obtained.

## 1. Introduction

Hybrid and cationic nanomaterials have been much explored in pharmaceutics for drug delivery [1,2], antimicrobial chemotherapy [3], dentistry [4], prosthetics [5], water treatment [6], and combination therapies against multidrug resistant pathogens [7,8]. These nanomaterials can have different shapes and compositions, yielding a variety of NPs and coatings.

The search for cationic NPs with potential as adjuvants for vaccines yielded many examples of polymer lipid [9,10,11,12,13,14,15,16,17], silica/cationic lipid [18,19], cationic polymer [20,21,22], gold/cationic polymer NPs [23], and iron-oxide/cationic polymer/antigen NPs [24]. Among the most important requirements of adjuvants for vaccines are biocompatibility and nanometric size [25].

Emulsion polymerization of methyl methacrylate (MMA), producing poly (methyl methacrylate) (PMMA), is well described in the literature [26]. In the presence of dioctadecyldimethylammonium bromide (DODAB) and poly (diallyl dimethylammonium) bromide (PDDA), emulsion polymerization of MMA yielded PMMA/DODAB/PDDA NPs [13,14]. These hybrid, PMMA-based NPs predominantly contained PMMA, a biocompatible polymer [27], displaying absence of cytotoxicity when tiny amounts of cationic components are used, as shown in this work.

Two different modes of interaction between lipids and polymers were previously disclosed: (1) Lipids phase-separating from polymers (low miscibility lipid/polymer); in this case, bilayer adsorption sometimes took place on the surface of polymer NPs [10,28,29,30]. (2) Lipids mixing well with the polymer such as DODAB and PMMA [9,11,13,15,31].

DODAB and PDDA in the medium used for MMA emulsion polymerization yielded antimicrobial core–shell cationic PMMA/DODAB/PDDA NPs, where DODAB remained in the PMMA core whereas PDDA surrounded the NPs as a cationic shell at low ionic strength [13,14].

The electrostatic attraction between anionic antigens and cationic nanostructures has been the basis for antigen/adjuvant assemblies, where nanometric size is implied in direct antigen delivery to lymph nodes. This avoids reactions at the injection site caused by the permanence of adjuvant/antigen complexes, allowing rapid antigen delivery to lymph nodes dendritic cells [32,33]. This work emphasizes the importance of cationic, biocompatible, and nano-sized polymeric particles for antigen presentation to the immune system. Here, PMMA/DODAB/PDDA NPs synthesized by emulsion polymerization of MMA, in the presence of PDDA and DODAB, were evaluated as adjuvants in vivo while carrying ovalbumin (OVA) as the model antigen.

## 2. Materials and Methods

### 2.1. Materials

MMA, PDDA, azobisisobutyronitrile (AIBN), NaCl, DODAB, chloroform, grade V ovalbumin, peroxidase-labeled streptavidin, and concanavalin A were from Sigma-Aldrich. PDDA molecular weight <100,000 g came as a 35% water solution. OVA stock solution (20 mg/mL) in Milli-Q water was purified by chromatography (polymyxin B columns; Pierce Biotechnology, Rockford, IL, USA), analyzed by the bicinchoninic acid (BCA) assay, and adjusted to 10 mg/mL. Al(OH)_3_ was from Sanofi-Synthelabo, RJ, Brazil. The NPs syntheses were performed in 1 mM NaCl solution. Milli-Q water was used for preparing all solutions and dispersions. For dialysis, a cellulose acetate bag with molecular weight cut-off around 12,400 g/mol was used. All other reagents were analytical grade and used without further purification.

### 2.2. Synthesis of NPs by Emulsion Polymerization Method

DODAB differs from surfactants by being a lipid. As such, the process for dispersing DODAB in the aqueous solution used as a reaction mixture requires a first step of preparing a DODAB chloroform solution so that a DODAB film can be obtained at the bottom of the glass assay tube by chloroform vaporization. This film in contact with the water solution will facilitate DODAB dispersion in the reaction mixture.

DODAB chloroform solution (10 mM) added to a glass tube (1.5 mL) was evaporated under nitrogen flux before adding 10 mL of 1 mM NaCl aqueous solution, 1 mL of 50 mg/mL PDDA, 0.20 mL of MMA 1.0 M followed by application of moderate nitrogen flux and AIBN addition (0.0036 g); polymerization occurred preferentially at 70–80 °C/1 h under periodic vortexing at 0.2 M MMA, 1.5 mM DODAB, 5 mg/mL PDDA [13,14]; the reaction mixture was extensively dialyzed until 5 µS/cm of water conductivity. These final concentrations in the reaction mixture were selected after systematic evaluation of the concentrations in the reaction mixture.

### 2.3. Determining Physical Properties of NPs by Dynamic Light-Scattering (DLS)

Dispersions were diluted 20 times for zeta-average diameter (Dz), zeta-potential (ζ), and polydispersity (P) DLS measurements using a Zeta-Plus Zeta-Potential Analyzer (Brookhaven Instruments Corporation, Holtsville, NY, USA), which was equipped with a 677 nm laser and dynamic light-scattering (DLS) at 90° for particle sizing. From fluctuations of scattered light intensity, the decay times of the fluctuations can be related to the diffusion constants and, therefore, to the sizes of the particles. Small particles moving rapidly cause faster decaying fluctuations than large particles moving slowly. The decay times of these fluctuations are determined in the time domain using a correlator. The fluctuating signal is processed by forming the autocorrelation function which decays exponentially with time so that the relaxation of the fluctuations is directly related to the decay constant (Γ). The decay constant is given by Equation (1):

Γ = *Dq*^2^ (1)

where *q* depends on the scattering angle, the wavelength of the laser light, and refractive index of the suspending liquid and *D* is the translational diffusion coefficient.

Hydrodynamic particle diameter (*D*z) is inversely related to *D* by the Stokes–Einstein Equation (2):

*D*z = *kT*/(3πη*D*) (2)

where *k* is the Boltzmann’s constant, *T* is temperature in Kelvin, η is the viscosity of the suspending liquid, and *D* is the diffusion coefficient. Mean diameters were obtained by fitting data to log-normal size distributions that do not discriminate between one, two, or more different populations and always consider all scattering particles as belonging to one single Gaussian population. For the size distribution data, fitting was performed by the apparatus software using the non-negatively constrained least-squares (NNLS) algorithm, which is a model-independent technique allowing multimodal distributions to be achieved [34].

In order to define a relative width of size distributions, the polydispersity P is given by Equation (3):

P = µ_2_Γ^2^ (3)

where μ_2_ is proportional to the variance of the “intensity” weighted diffusion coefficient distribution and carries information on the width of the size distribution. Polydispersity (P) has no units. It is close to zero (0.000 to 0.020) for monodisperse or nearly monodisperse samples, small (0.020 to 0.080) for narrow distributions, and larger for broader distributions. ζ was determined from electrophoretic mobility (µ) in 1 mM NaCl and the Smoluchowski equation (Equation (4)):

ζ = μη/ε(4)

where η is the medium viscosity and ε the medium dielectric constant.

### 2.4. Determining Solids Content, PDDA, and DODAB Concentration in NPs

Solids content (mg/mL) was obtained from lyophilizing and weighing 1 mL NPs dispersions. [PDDA] in NPs ([PDDA]_p_) was obtained by subtracting free [PDDA] from added [PDDA]. Centrifuging dispersions (1 h/10,000 rpm) and pelleting NPs allowed the determination of [PDDA] in the supernatant from conductance, since [PDDA] and conductance displayed a linear relationship. During dialysis, PDDA attracted water by osmosis; increased volume after dialysis required measuring final volume after dialysis for precise calculations of final concentrations.

[DODAB] in NPs [DODAB]_p_ was obtained from 1 mL dialyzed dispersion after centrifuging (1 h/10,000 rpm), supernatant withdrawing, and halide microtitration [35] in the supernatant containing Br^−^ and Cl^−^ from DODAB and PDDA, respectively. Subtracting supernatant [PDDA] (previously determined by conductimetry) from [halides] yielded the supernatant [DODAB] required for determining [DODAB]_p_.

### 2.5. Determining NaCl Concentration Effect on NPs Dz

The core/shell NPs structure was determined as previously described [12,13]. Dialyzed NPs were centrifuged, the supernatant was removed, and the pellet was resuspended in pure water before 1:40 dilution in aqueous NaCl solutions (1–75 mM NaCl) and 30 min interaction for determining Dz by DLS. The core diameter was the minimal Dz (Dz_min_) at collapse of the PDDA shell (salt screened PDDA charges); shell thickness was Dz_0_–Dz_min_, where Dz_0_ is Dz in pure water.

### 2.6. Evaluation of NPs/OVA Interaction

The dialyzed and treated NPs contained 7.0, 0.32, and 0.7 mg/mL PMMA, DODAB, and PDDA, respectively; NPs (0.071 mL; 0.05 mg/mL PDDA) interacted 1 h with OVA stock solution over a [OVA] range before DLS measurements or [OVA] determination in the supernatants [36]. Adsorbed [OVA] was obtained from added minus free [OVA].

### 2.7. Determination of NPs Cytotoxicity by MTT Assay

L929 fibroblast and J774A.1 macrophage from American Type Culture Collection were cultured according to standard protocols (90% humidity, 5% CO_2_, 37 °C), RPMI-1640/5% fetal bovine serum (FBS), 1 unit/mL penicillin-streptomycin, and 2 mM l-glutamine. NPs diluted in the same medium (0.1 mL) interacted with plated cells (10,000 cells/well; 12 h incubation) after withdrawing 0.1 mL of culture medium for incubation in a humidified CO_2_ incubator for 3 and 24 h before determining the in vitro NPs’ cytotoxicity using 3-(4,5-dimethylthiazol-2-yl)-2,5-diphenyl tetrazolium bromide (MTT). MTT (5 mg/mL in PBS) was filtered for sterilization and removal of insoluble residues before adding 10 μL of MTT solution per well containing 100 µL of the cells and NPs at different NPs concentrations. After incubation (37 °C/2 h), supernatant withdrawing, and mixing of adhered cells with 100 µL isopropanol solution in 0.04 N HCl, absorbance at 570 nm was determined on an ELISA reader; 100 % cell viability was given by cells in culture medium only.

### 2.8. Immunization Protocol

Four groups of five BALB/c male mice each were injected subcutaneously at two sites on the base of the tail with 0.2 mL/animal. Solutions or dispersions used for injection were: (1) pure water; (2) 0.1 mg/mL OVA; (3) 0.1 mg/mL OVA/0.1 mg/mL Al(OH)_3_; (4) 0.1 mg/mL OVA in NPs with 0.52, 0.05, and 0.04 mg/mL of PMMA, PDDA, and DODAB, respectively. A booster (same dose/21 days post immunization) was injected. Guidelines for experiments with animals were from Instituto Butantan’s Committee of Ethics on Animal Research (protocol 7912280219).

### 2.9. Determining OVA-Specific IgG1 and IgG2a

Blood from mice was collected from the ophthalmic plexus 14, 21, and 28 days after immunization yielded serum which was individually analyzed by the indirect enzyme-linked immunosorbent assay (ELISA); 96-well microtiter plates (Costar Corning Inc., Cambridge, MA, USA) were coated overnight at 4 °C with 100 μL 10 μg/mL OVA solution in 0.01 M phosphate buffer saline, pH 7.2. The wells were blocked (3% gelatin/PBS/2 h), incubated with serially diluted serum (1 h/37 °C), added of 100 μL goat anti-mouse biotin-conjugated IgG (1:1000) or IgG2a (1:500) (Southern Biotechnology Associates, AL, USA), and incubated (1 h/37 °C) again before adding peroxidase-labeled streptavidin (100 μL/1:3000 dilution) for 1 h incubation (at 37 °C), washing three times after each incubation step with PBS/0.05% Tween 20. Finally, 100 μL 1 mg/mL OPD solution and H_2_O_2_ (1 μL/mL) in 0.1 M citrate-phosphate (pH 5.0) were added per well for incubation (15 min/room temperature) before stopping the reaction with 0.05 mL 2 M H_2_SO_4_ per well. Absorbance was determined at 492 nm and results were expressed as mean values ± mean standard deviation. IgG1 and IgG2a primary responses were at 1/512 and 1/8; secondary responses, at 1/32696 and 1/256 dilutions, respectively. Results from different groups were compared using two-way variance analysis (ANOVA) and Tukey’s test. *p*-values < 0.05 were significant. Separate statistics for IgG1 or IgG2a were performed.

### 2.10. Determining DTH from Footpad Swelling

Mice at day 5 post immunization were injected at the left-hind footpad with OVA, denatured by heating, in saline (2 mg/mL; 0.03 mL; 0.03 mL saline as control). Footpad swelling 24 h thereafter was measured with a micrometer as the difference between left and right footpads’ swelling per animal and given as mean values ± mean standard deviation.

### 2.11. Culturing Spleen Cells for Cytokines Analysis

Two weeks after the second injection, spleen cells from five mice were gathered in RPMI 1640 with 10 mM HEPES, 50 μM 2-mercaptoethanol, 216 mg/L l-glutamine, and 5% FBS. Suspensions (12 × 10^6^ cells/mL) distributed in tissue culture plates were incubated with medium containing 250 μg/mL OVA in a humidified CO_2_ incubator for 72 h: medium only or 2.5 μg/mL concanavalin A (con A) were negative and positive controls, respectively. Thereafter, plates were centrifuged (5 min/1500 rpm) and the supernatants were collected for determining IL-2, IL-4, IL-10, and IFN-γ (sandwich ELISA kit; catalog #88-7711-44 Thermo Fisher Scientific). Detection limits were 0.03 (IL-10), 0.002 (IL-2), 0.004 (IL-4), and 0.015 ng/mL (IFN-γ); secretions are expressed as mean for two assays ± mean standard deviation.

## 3. Results and Discussion

### 3.1. Synthesis of PMMA/DODAB/PDDA NPs

The synthesis of PMMA NPs in the presence of DODAB or PDDA yielded poor conversion of MMA into PMMA using potassium persulfate as the initiator [11] or PDDA only, as the emulsifier [12]; conversion increased substantially with the AIBN initiator and DODAB/PDDA emulsifiers [13,14]. However, systematic evaluation of the effect of [MMA] or [PDDA] on the conversion or physical properties of the NPs dispersions had not been performed before.

The systematic effect of MMA and PDDA concentrations on yield of PMMA/DODAB/PDDA NPs synthesized using AIBN as the initiator is on Table 1. This table shows solid contents and yield for the synthesis of PMMA/DODAB/PDDA NPs (at 1.5 mM DODAB, 5 mg/mL PDDA, and 0.0036 g AIBN). The increment in MMA concentration increased the solid contents since MMA molecules available for polymerization increased. However, the yield decreased as [MMA] increased, meaning that not all monomers in the reaction mixture polymerized. This behavior differed from the one observed in the synthesis of PMMA/PDDA NPs in the absence of a surfactant. Under these conditions, over the same range of MMA and PDDA concentrations employed in this work, solids content fluctuated between 3.1 and 5.8 mg/mL, in contrast to the higher values achieved in Table 1, namely, 11–36 mg/mL [14]. The use of DODAB as an additional emulsifier in the synthesis of these NPs increased the mass of PMMA synthesized from MMA; this behavior was observed for all the tested MMA concentrations. However, using MMA concentrations above 0.2 M caused the appearance of precipitates during the synthesis. Increasing [MMA] decreased the yield, possibly because DODAB and PDDA concentrations were not enough to stabilize all the MMA droplets in the reaction mixture. During the synthesis of PMMA/PDDA NPs under the same conditions but in the absence of DODAB, the maximum yield was 25% at 0.05 M MMA, showing the essential role of DODAB as an emulsifier [14].

**Figure 1:**
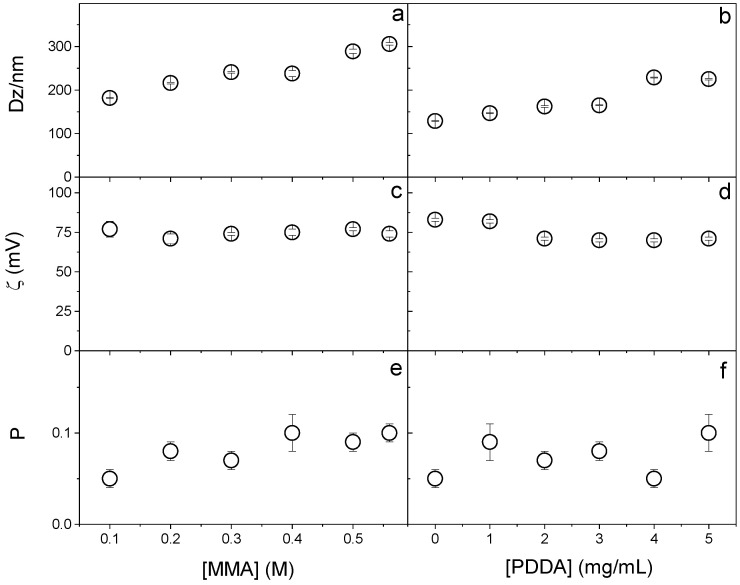
Effect of MMA or PDDA concentrations on (**a**,**b**) hydrodynamic diameter (Dz), (**c**,**d**) zeta-potential (*ζ*), and (**e**,**f**) polydispersity (P). All dispersions were synthesized by the emulsion polymerization method using 1.5 mM of DODAB and 0.0036 g of AIBN initiator. To evaluate the effect of MMA concentration, the synthesis was performed at 5 mg/mL of PDDA and to evaluate the effect of PDDA, 0.2 M MMA was used. Data points are mean values ± the mean standard deviation.

The effect of MMA and PDDA concentrations on the physical properties of NPs obtained in the presence of DODAB 1.5 mM was explored to establish the best conditions for synthesizing PMMA/DODAB/PDDA NPs (Figure 1). Increasing MMA concentration increased D_Z_ (Figure 1a), probably due to increasing the growth in the mass of PMMA in the NPs, which did not affect the zeta-potential (Figure 1c) and slightly increased polydispersity over a range of [MMA] at 5 mg.mL^−1^ PDDA (Figure 1e).

**Figure 2:**
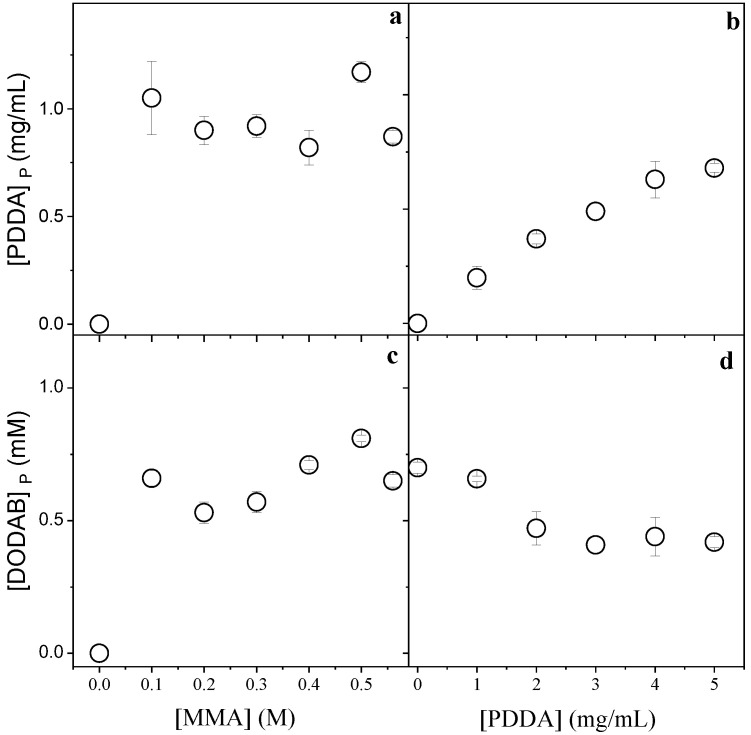
Effect of MMA and PDDA concentrations used in the NPs synthesis on [PDDA]_P_ (**a**,**b**) and [DODAB]_P_ in the PMMA/DODAB/PDDA NPs (**c**,**d**). All dispersions were synthesized by the emulsion polymerization method using 1.5 mM of DODAB and 0.0036 g AIBN. The effect of [MMA] on incorporation of the cationic components was performed at 5 mg/mL PDDA, whereas the effect of [PDDA] on incorporation of the cationic components was performed at 0.2 M MMA. These determinations were carried out after dialysis.

When increasing the PDDA concentration, Dz increased (Figure 1b) because more PDDA was incorporated into the NPs (as clarified in Figure 2); the zeta-potential remained constant (Figure 1d) and the polydispersity changed from 0.05 at 0 [PDDA] to 0.100 at 5 mg/mL [PDDA] (Figure 1f).

Figure 2 displays the effect of MMA or PDDA concentrations used in the synthesis of PMMA/DODAB/PDDA NPs on the concentration of DODAB and PDDA incorporated in the NPs.

Figure 2a,c show that maximal incorporation of the cationic components was reached from 0.1 M MMA. Figure 2b shows that the incorporation of PDDA increased with [PDDA]. Figure 2d shows that the incorporation of DODAB decreased with [PDDA]. The increment in PDDA concentration leads to an increase in mechanically immobilized PDDA in the NPs and to a simultaneous decrease in immobilized DODAB. The two cationic components, PDDA and DODAB, competed for the PMMA matrix. Increasing the PDDA concentration during NPs synthesis, the incorporated [DODAB] in the PMMA matrix decreased (Figure 2d).

From the data shown in Table 1 and Figure 1 and Figure 2, 0.2 M MMA was selected for the synthesis of NPs to be used as adjuvants. At this concentration, no precipitates occurred and relatively high solids content (14 mg/mL) and yield (70%) were obtained. [PDDA] was selected as 5 mg/mL because this was the lowest concentration where maximal PDDA incorporation occurred.

### 3.2. Properties and Cytotoxicity of PMMA/DODAB/PDDA NPs

First of all, PMMA/DODAB/PDDA NPs prepared with the AIBN initiator were treated to eliminate free DODAB and PMMA, assuring antigen adsorption to NPs only; treatment was pelleting dispersions, withdrawing and discarding supernatants, and resuspending the pellet in pure water. Table 2 shows the NPs’ properties before and after treatment. Although main NPs’ properties were not affected, the treatment reduced the conductance of the dispersion to practically zero due to the removal of free PDDA. PMMA density (1.18 g.cm^−3^) [37], mean NP volume (5.3 × 10^−15^ cm^3^), and mean mass per NP (6.2 × 10^−12^ mg) yielded 1.3 × 10^12^ particles/mL with 7.0 mg/mL PMMA in this stock dispersion and precise knowledge of particle number density, [PDDA]_P,_ and [DODAB]_P_ (Table 2).

**Figure 3:**
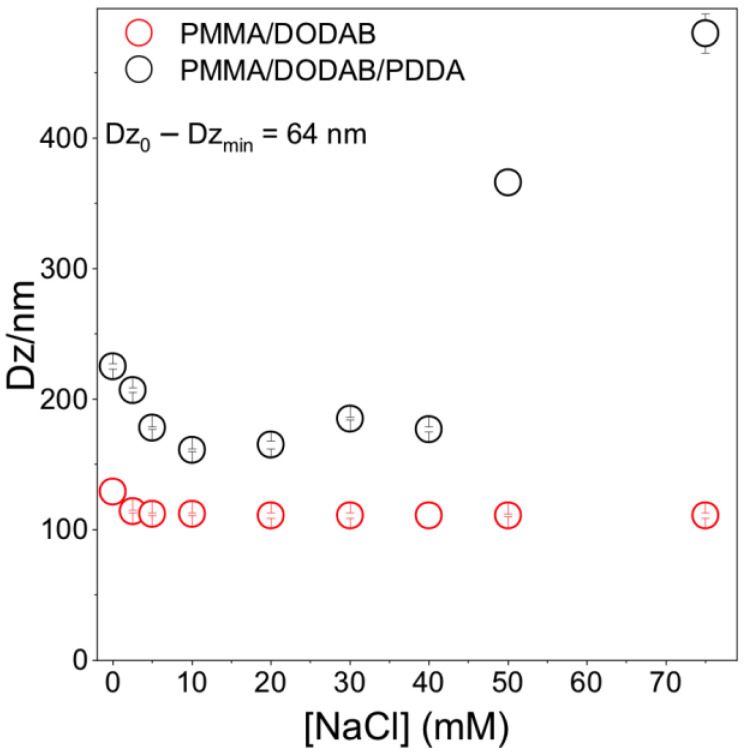
Effect of [NaCl] on PMMA/DODAB/PDDA or PMMA/DODAB NPs Dz. At [NaCl] = 0, Dz_0_ =225 ± 2 nm. At 10 mM NaCl, Dz_min_ =161 ± 1 nm yielding 161 ± 1 nm core and 64 nm shell.

Core–shell dimensions in the NPs were evaluated using a method based on determining Dz as a function of [NaCl] [12]. When PDDA is fully charged (pure water), extended PDDA chains in the outer NP layer yielded the thickest shell. The NaCl screening effect on PDDA charges reduced shell thickness in comparison to the one in pure water. Figure 3 shows that the shell thickness at [NaCl] = 0 is 64 nm. Shell thickness for similar PMMA/PDDA NPs (in absence of DODAB) was 80 nm [12]; here, 64 nm seems consistent with DODAB in the PMMA core, yielding a thinner PDDA shell due to PDDA electrostatic repulsion by DODAB in the core.

For [NaCl] > 40 mM, Dz increased with [NaCl] (Figure 3). Bridging flocculation has been observed for colloids with outer PDDA layers [16,38]. For PMMA/DODAB NPs, no bridging flocculation occurred; Dz was constant with increasing [NaCl] (Figure 3). PMMA/DODAB NPs high colloidal stability was due to embedded DODAB molecules in the PMMA core unaffected by outer NaCl.

**Figure 4:**
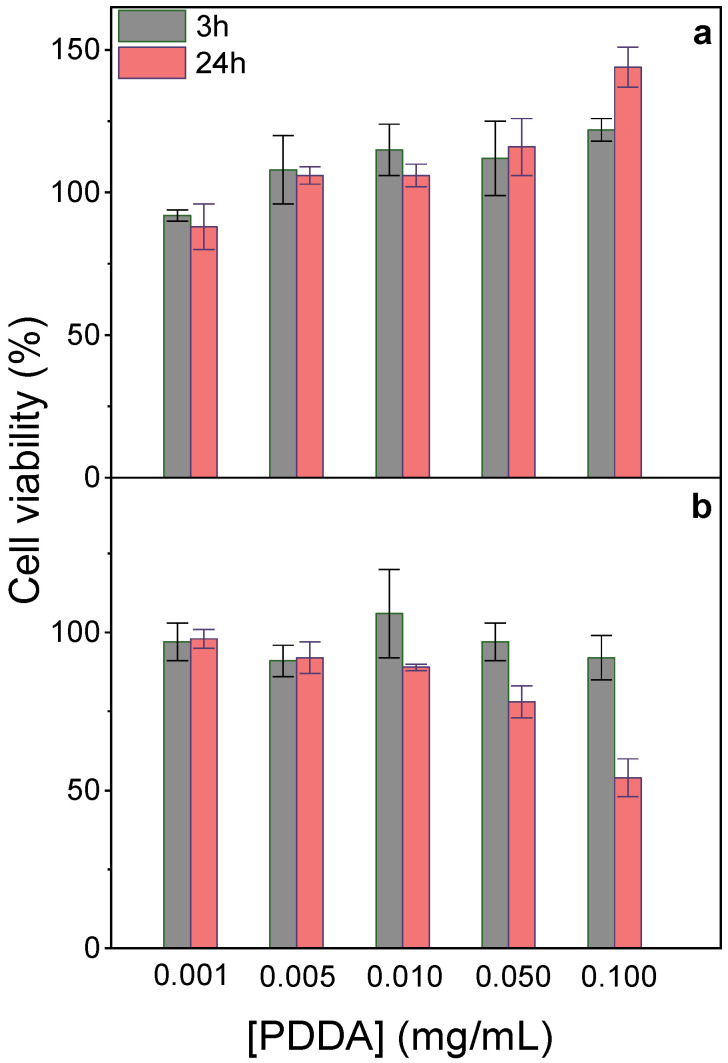
Effect of immobilized PDDA concentration on NPs on L929 fibroblasts (**a**) or J774A.1 macrophages viability (%) after 3 or 24 h interaction (**b**). The stock dispersion contained 0.7 mg/mL immobilized PDDA.

At 0.05 mg/mL PDDA in PMMA/DODAB/PDDA NPs, 100% viability was observed against fibroblasts (Figure 2a); 80% viability after 24 h interaction was obtained against macrophages (Figure 4b). Cationic polyelectrolytes usually improve fibroblasts adhesion and proliferation on surfaces [39,40], in good agreement with our data (Figure 4a). Macrophages sensitivity to PDDA was observed for colloids with an outer PDDA layer [41]; at 0.1 mg/mL PDDA, fibroblasts exhibited major holes on cell membranes yielding 25% viability after 3 h interaction [20]. Free PDDA is very cytotoxic, whereas toxicity of immobilized PDDA is much more controllable. Macrophages interacting for 48 h with assemblies of DODAB/carboxymethylcellulose/PDDA containing 0.025 mg/mL PDDA were only 30% viable [41], whereas NPs containing 0.050 mg/mL immobilized PDDA yielded 80% macrophages viability for 24 h of interaction (Figure 4b). Mechanical PDDA immobilization in NPs prevented its complete interaction with the cells, avoiding the cell lysis related to free/penetrating cationic polymer chains. PDDA immobilization improved its biocompatibility by reducing cytotoxicity in comparison to free PDDA. In contrast to fibroblasts, macrophages were more sensitive to the cationic NPs, possibly due to their phagocytic ability. They avidly take up cationic NPs by direct translocation, endocytosis, or other mechanisms [21].

In fact, several pieces of evidence suggested that PDDA dose-dependent cytotoxicity can be reduced upon PDDA immobilization onto NPs. For example, against bacteria and fungus, immobilized PDDA was much less toxic than free PDDA [12]. The dose-dependent cytotoxicity of poly-cations, e.g., poly-lysine, poly-ethylene-imine, and PDDA, evaluated by MTT assay or lactate–dehydrogenase release, yielded 30% viability after 24 h interaction of fibroblasts with 0.1 mg/mL PDDA [42]. When reducing free PDDA dose to 0.01 mg/mL, about 100% fibroblasts remained viable [20,42].

### 3.3. OVA/NPs Interaction

**Figure 5:**
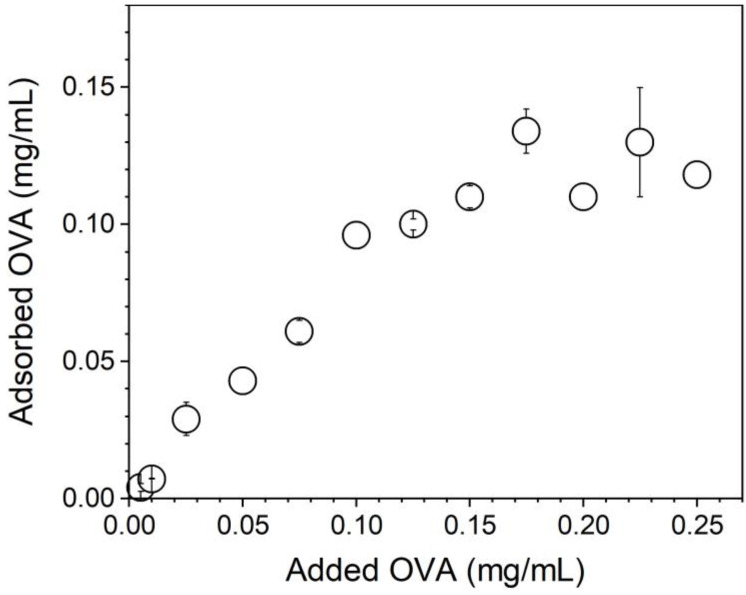
OVA adsorption isotherm onto PMMA/DODAB/PDDA NPs in Milli-Q water.

Figure 5 shows that OVA adsorption increased linearly with the added [OVA] until maximal OVA adsorption at 0.10–0.12 mg/mL OVA from 0.10 mg/mL of added OVA. Above 0.1 mg/mL of added [OVA], adsorption remained constant and equal to 0.1–0.12 mg/mL OVA. The lowest [OVA] added to obtain maximal adsorption was 0.1 mg/mL (Figure 5).

**Figure 6:**
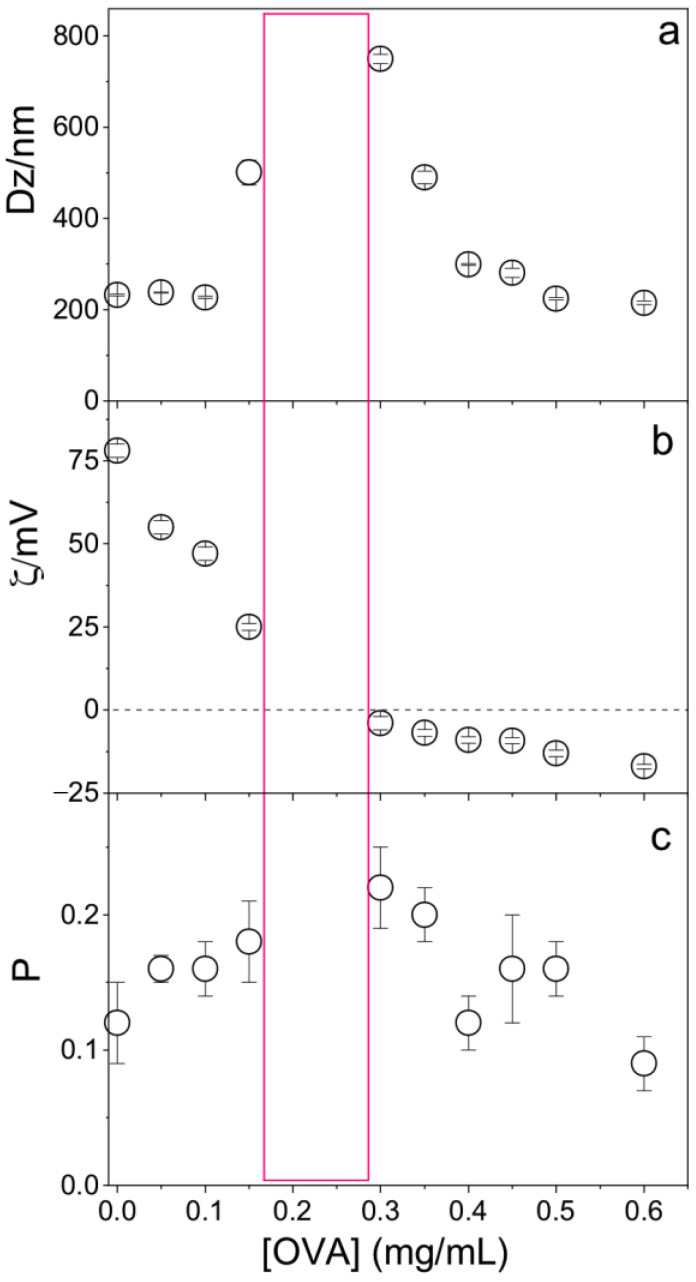
Effect of ovalbumin (OVA) concentration on (**a**) the mean hydrodynamic diameter (Dz), (**b**) zeta-potential (ζ), and (**c**) polydispersity (P) of PMMA/DODAB/PDDA NPs. The red rectangle indicates the zone where the dispersion was not colloidally stable and DLS measurements were not reliable.

OVA concentration affected the physical properties of the PMMA/DODAB/PDDA NPs; at [OVA] = 0.17 mg/mL, zeta-potential was zero and NPs/OVA displayed poor colloidal stability with the occurrence of precipitates and poor DLS measurements (Figure 6). Outside this region, a good colloidal stability allowed for reliable DLS measurements. At 0.1 mg/mL OVA, cationic NPs displayed *ζ* = 45 ± 2 mV, P = 0.15 ± 0.02, and Dz = 242 ± 4 nm; this concentration of OVA was selected for the evaluation of NPs as adjuvants.

The colloidal stability after the NPs’ synthesis and 72h dialysis was evaluated from measurements of the physical properties for the NPs at two time points: 1 and 6 h. For the NPs with OVA, a similar control was conducted revealing no changes in physical properties. All PMMA/DODAB/PDDA/OVA NPs injected in vivo were prepared and used 1 h after preparation.

The bare NPs (Dz = 217 ± 1 nm) adsorbed OVA, yielding 25 nm of an adsorbed layer (Figure 6). Silica/DODAB dispersions (Dz = 304 ± 2 nm) yielded Dz = 324 ± 8 nm at maximal adsorption with bovine serum albumin (BSA) or 20 nm BSA thickness [19]. It is interesting to compare the relative amount of protein that can be adsorbed onto a supported cationic bilayer and onto a cationic polymer shell surrounding solid NPs. Taking into account the 65.5 kDa (BSA) and 45 kDa molecular weight (OVA), if the same number of molecules had been adsorbed in both cases, at maximal adsorption, one would expect to find a thicker BSA layer than the OVA layer on each NP. The experimental result yielded a thicker OVA layer than the BSA layer, showing that the number of OVA molecules adsorbed onto the PMMA/DODAB/PDDA NPs was higher than the number of BSA molecules adsorbed onto the silica/DODAB NPs at maximal adsorption for both systems. Possibly, different surfaces of the core–shell and silica/DODAB NPs interacted differently with the proteins. Whereas the former presented to the protein a cationic polymer layer with several protrusions and indentations due to the extended polymer chains, the latter were smooth cationic surfaces as provided by the adsorption of a DODAB cationic bilayer onto each silica NP. The total surface area for adsorption is higher in the former than in the latter cationic surface. The polymer shell was a more efficient rough surface for protein adsorption. Consistently, the maximal adsorption values obtained in both cases show that the adsorbed protein layer is indeed thicker at maximal adsorption for the core–shell NPs than for the silica/DODAB NPs. Adsorption is 2.5× higher on the core–shell than on the silica/DODAB NPs (Figure 5). For the polymer surface upon adding 0.100 mg/mL OVA, maximal adsorption was achieved at 0.100 mg/mL (Figure 5), whereas upon adding 0.050 mg/mL, maximal adsorption was achieved at 0.020 mg/mL [19].

### 3.4. PMMA/DODAB/PDDA NPs as Adjuvants

**Figure 7:**
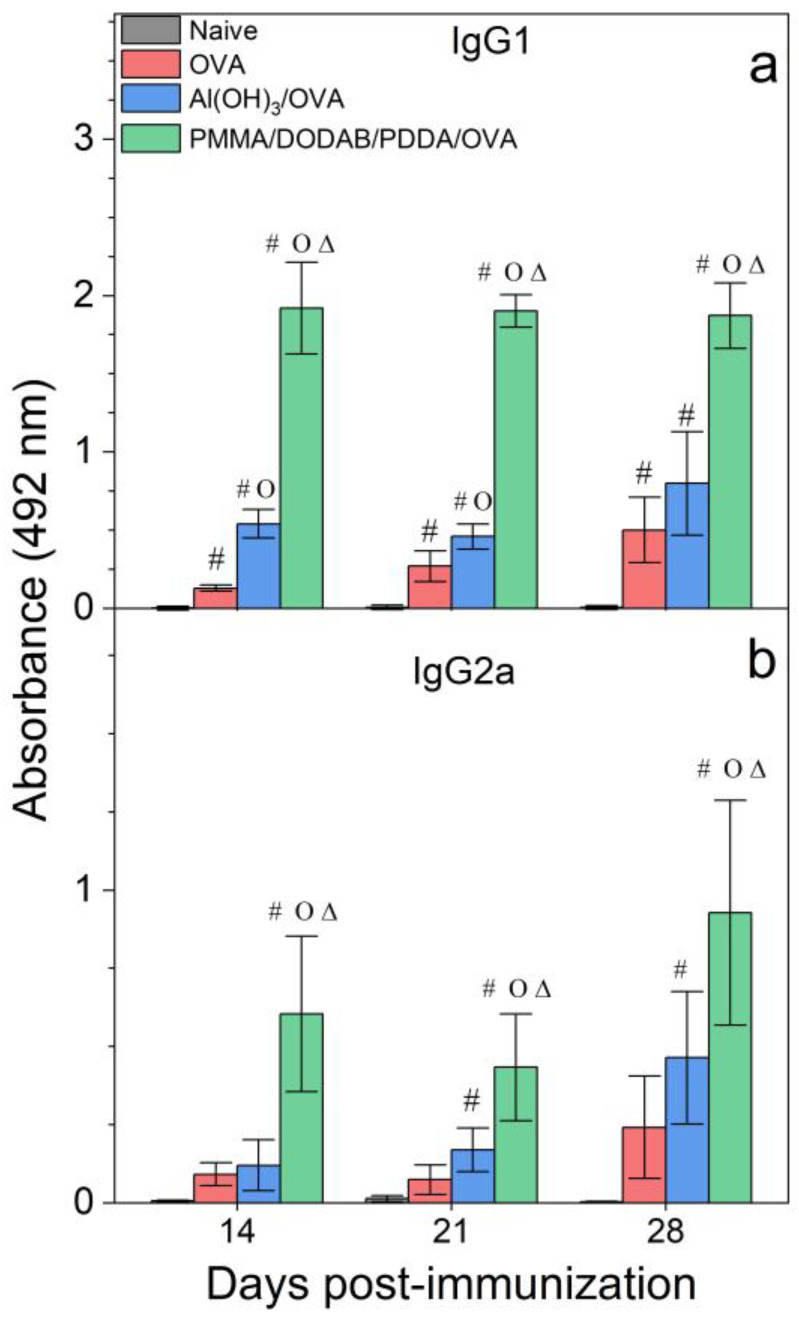
Humoral responses induced by OVA, Al(OH)_3_/OVA, or PMMA/DODAB/PDDA/OVA NPs. Mean absorbance at 492 nm ± standard deviation related to anti-OVA IgG1 (**a**) and IgG2a (**b**) antibodies production was determined over time after immunization of BALB/c mice from sera collected on days 14 and 21 post immunization (primary response) or on day 28 (secondary response). Primary IgG1 production was determined at 1/512 serum dilution, whereas the secondary one was obtained at 1/32,696 dilution. Primary IgG2a production was determined at 1/8 serum dilution, whereas the secondary one was obtained at 1/256 dilution. *p* < 0.05 compared to the naive group (#), *p* < 0.05 compared to the OVA group (o), *p* < 0.05 compared to the Al(OH)_3_/OVA group (∆).

**Figure 8:**
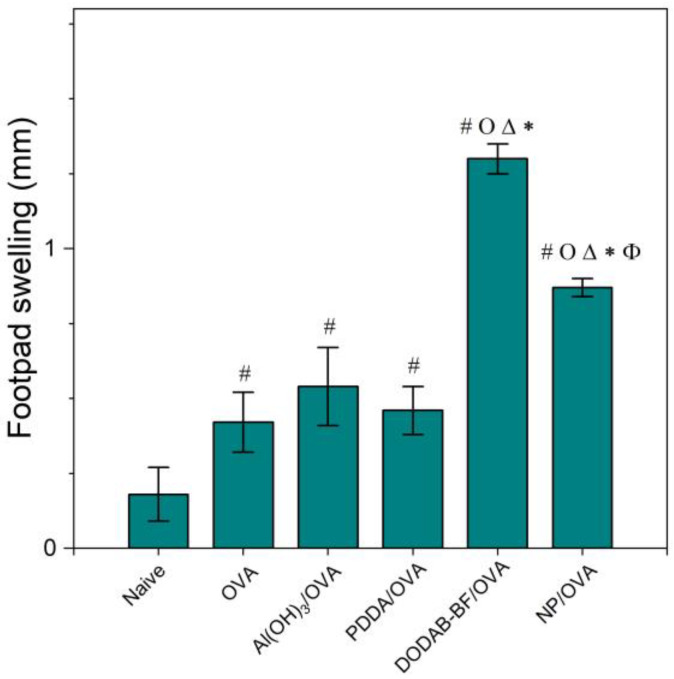
Mean footpad swelling ± standard deviation related to the delayed-type hypersensitivity reaction of BALB/c mice immunized with OVA, Al(OH)_3_/OVA, PDDA/OVA [20], DODAB-BF/OVA [44], or PMMA/DODAB/PDDA NPs carrying OVA (NP/OVA). *p* < 0.05 compared to the naive group (#), *p* < 0.05 compared to the OVA group (o), *p* < 0.05 compared to the Al(OH)_3_/OVA group (∆), *p* < 0.05 compared to the PDDA/OVA group (*), or *p* < 0.05 compared to the DODAB-BF/OVA group (ϕ). The controls for PDDA/OVA and DODAB BF/OVA previously published (naïve, OVA) were very similar to those shown here for NP/OVA.

**Figure 9:**
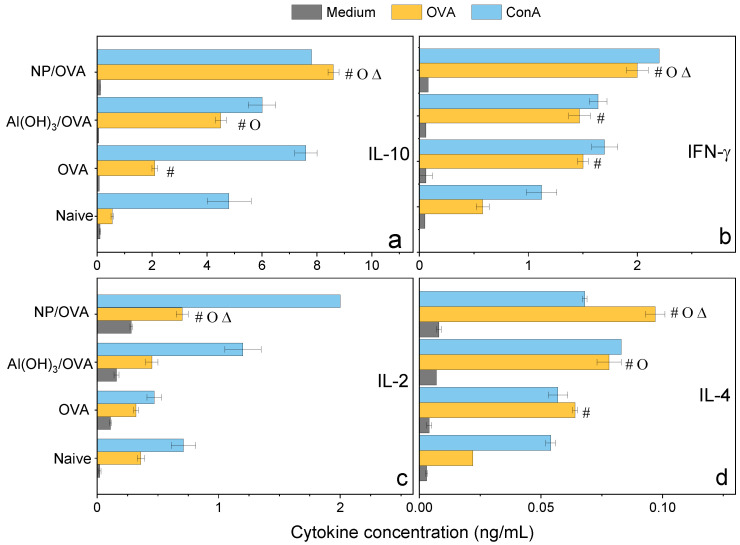
Cytokines profile (ng/mL) from the cultured spleen cells of BALB/c mice, naïve or immunized with OVA, Al(OH)_3_/OVA, PMMA/DODAB/PDDA/OVA (NP/OVA). *p* < 0.05 compared to the naive group (#), *p* < 0.05 compared to the OVA group (o), *p* < 0.05 compared to the Al(OH)_3_/OVA group (∆). (**a**) IL-10, (**b**) IFN-γ, (**c**) IL-2, (**d**) IL-4.

The adjuvant activity of NPs/OVA in vivo was evaluated from OVA-specific IgG1 and IgG2a production (Figure 7), the delayed type hypersensitivity (DTH) reaction from footpad swelling in mice (Figure 8), and determination of IL-2, IL-4, IL-10, and IFN-γ production by the spleen cells of the immunized mice in culture (Figure 9).

Adjuvants as Al(OH)_3_ are of choice for implementing the antigen-specific production of IgG1 [43], whereas immune-adjuvants as vesicles or bilayer-fragments of the cationic lipid DODAB (DODAB BF) usually implement the antigen-specific production of IgG2a [17,44,45,46]. In Figure 7, NPs/OVA revealed their potent activity as inducers of humoral immune response in the production of IgG1, surpassing by about four times the primary response induced with the classical Al(OH)_3_ adjuvant. The production of IgG2a (Figure 7) and the DTH reaction (Figure 8) followed similar patterns with the NPs, yielding much higher figures than the Al(OH)_3_ adjuvant, a poor inducer of cellular immune responses [42].

DODAB BF/OVA and PMMA/DODAB/PDDA/OVA yielded a DTH response; Al(OH)_3_/OVA or PDDA/OVA did not elicit a DTH response as previously reported in reference [20] (Figure 8). The NPs/OVA yielded both humoral and cellular immune responses (Figure 7 and Figure 8).

Three different cationic nanoparticles have their performance as adjuvants compared in Table 3. They were: DODAB BF [44], PDDA/OVA NPs [20], and PMMA/DODAB/PDDA NPs (this work). OVA-specific IgG1 and IgG2 production and footpad swelling (FS) corresponding to the DTH reaction to OVA alone or carried by cationic NPs showed that DODAB BF were the most potent ones to induce DTH and IgG2a. PMMA/DODAB/PDDA NPs represented the most potent adjuvant to induce IgG1 response. The moderate increase in Th-1 response induced by the PMMA/DODAB/PDDA NPs could be explained by an eventual ability of cationic NPs to cross the cytoplasmic membrane by direct translocation, escaping endocytosis. Fluorescent giant vesicles made of phospholipid bilayers interacting with fluorescent cationic amidine polystyrene NPs of 20 nm diameter allowed the visualization of NPs adhesion and permeabilization of the model membrane to polymers of high molecular weight, indicating the formation of transient pores across bilayers and NP translocation [47]. In another study using molecular dynamics simulations, a model cell membrane allowed direct translocation of cationic gold nanoparticles (AuNPs) as well as HIV-1 Tat peptides; thereafter, the membrane resealed itself within a microsecond [48].

The three cationic adjuvants shown in Table 3 may have their adjuvant characteristics understood as the degree to which antigen delivery to the cytoplasm does or does not avoid endocytosis. Which factors would contribute to cytoplasm delivery of the carried antigen? Essentially, major factors would be direct translocation and the antigen/adjuvant strength of binding. The behavior of DODAB BF/OVA may have resulted from direct translocation and release of the antigen in the cytoplasm plus a relatively weak strength of binding between DODAB BF and antigen; this would explain the predominance of the Th-1 response. In contrast, PDDA/OVA behavior yielding predominance of Th-2 response could be explained by a high binding strength between PDDA and OVA in an entangled assembly and/or poor frequency of direct translocation with predominant internalization by endocytosis. The PMMA/DODAB/PDDA NPs would possibly become internalized by both routes (endocytosis and direct translocation) with the occurrence of an intermediate strength of binding between OVA and immobilized PDDA, so that some OVA molecules would be released from the NPs in the cytoplasm; overall, the Th-1 and Th-2 responses would occur for this system (Table 3).

Confirmation of the mixed Th-1/Th-2 response was obtained by determining the cytokines profile induced in immunized mice 35 days after immunization and evaluated from cultured spleen cells production (Figure 9). At this point in time, one should expect that too high responses (either Th-1 or Th-2) would have been dampened by the secretion of a regulatory cytokine such as IL-10 so that levels of this molecule would reflect down-regulation of too high Th-1 or Th-2 responses. Indeed, levels of IL-10 were very high and even higher than those elicited by Al(OH)_3_ (Figure 9). IL-10 is produced by a diversity of cell types, including CD8^+^, CD4^+^, regulatory T cells, γδ-T cells, NK cells, NK-T cells, B cells, dendritic cells, eosinophils, mast cells, and monocytic cells; it is a cytokine with several pleiotropic effects in immunoregulation and inflammation [49,50,51]. IL-10 is an anti-inflammatory cytokine that downregulates the expression of Th-1 and Th-2 cytokines, MHC class II, and co-stimulatory B7-1/B7-2 molecules [49,50,51]. It maintains the balance of the immune response, allowing the clearance of infection while minimizing damage to the host [49,50]. The consequence of this activity, however, is that IL-10 can contribute to chronic infection [52].

The relatively low levels of IFN-γ and IL-2 determined in the spleen cells of mice immunized with the NPs/OVA at day 35 might reflect the regulatory activity of IL-10, although levels of these cytokines were still higher than those for the other groups (Figure 9).

IFN-γ enhances immune function by promoting the growth of Th-1 cells while inhibiting Th-2 cell growth and enhancing NK cytotoxic activity [49]. Furthermore, IFN-γ induces switching to IgG2a production by B cells and potentiates antigen presentation by activating dendritic cells and inducing MHC class I and class II gene expression on the surface of target cells [49]. IFN-γ is the major activator of macrophages, primarily through the induction of superoxide, nitric oxide production, and natural resistance-associated macrophage protein (NRAMP) gene expression [49,53]. At 6 days after immunization of mice with 18kDa-hsp antigen of *Mycobacterium leprae* presented by DODAB BF, 23 ng/mL IFN-γ and 0.6 ng/mL IL-10 secretions by cultured lymph node cells were determined [17]. In the present work, at day 35 after immunization with NPs/OVA, 2 ng/mL IFN-γ and 9.0 ng/mL IL-10 secretions by cultured lymph node cells were verified (Figure 9). Possibly the regulatory and dampening effect of this large secretion of IL-10 might be responsible for the low level of IFN-γ.

IL-2 is a proliferation factor of T cells [54]; the stimulation of naive CD4^+^ T cells differentiation into Th-1 and Th-2 lymphocytes was reported [49,55]. IL-2 quantification for NPs/OVA yielded 0.8 ng/mL, about double the value determined for Al(OH)_3_/OVA, suggesting that the double response induced by NPs/OVA (Th-1 and Th-2 types) required induction by doubled secretion of IL-2 (Figure 9).

Differentiation of naïve CD4^+^ T cells into Th-2 cells requires signaling through the T cell receptor and an appropriate cytokine environment; IL-4 is critical for Th2 differentiation [49,56]. In our cytokines profile, IL-4 secretion induced by NPs-OVA was 0.1 ng/mL and even higher than the IL-4 secretion induced by Al(OH)_3_/OVA, 0.07 ng/mL (Figure 9), agreeing with the compared IgG1 response shown for both systems (Figure 7a). The NPs/OVA are indeed better Th-2 inducers than Al(OH)_3_/OVA. Our results also indicate that the formulation NPs/OVA was a better inductor of OVA-specific Th-1/Th-2 responses when compared with Al(OH)_3_/OVA or OVA alone.

## 4. Conclusions

Cationic and biocompatible NPs of PMMA/DODAB/PDDA with Dz = 217 ± 1 nm, ζ = 73 ± 1 mV, and P = 0.12 ± 0.01 were investigated for their adjuvant properties while carrying the model antigen OVA. In addition, this system was compared with other cationic NPs and their immune responses in order to gain further insight into factors that determine Th-1 or Th-2 responses for cationic NPs. Two major factors were pointed out: (1) strength of adjuvant/antigen binding; (2) intracellular traffic with direct translocation across the cell membrane implying Th-1 response. DODAB BF/OVA, as Th-1 immune response inducers, translocated across the membrane to the cytoplasm releasing OVA; PDDA/OVA, as Th-2 inducers, did not release the protein in the cytoplasm requiring internalization by endocytosis; PMMA/DODAB/PDDA/OVA NPs were internalized by both routes, displaying both Th-1 and Th-2 responses. Among the three cationic systems useful as adjuvants, the highest biocompatibility occurred for the PMMA/DODAB/PDDA NPs, since they were based mostly on PMMA, a biocompatible polymer.

## Figures and Tables

**Table 1:** Effect of methyl methacrylate concentration MMA on the solids content (mg/mL) and on the yield of the conversion of MMA into poly (methyl methacrylate) (PMMA). The dispersions of PMMA, dioctadecyl dimethylammonium bromide (DODAB) and polydiallyldimethylammonium bromide (PDDA) as PMMA/DODAB/PDDA nanoparticles (NPs) were synthesized using 1.5 mM of DODAB, 5 mg/mL of PDDA, and 0.0036 g azobisisobutyronitrile (AIBN). The data refer to samples just after synthesis and before dialysis.

[MMA]/M	Solids Content/mg mL^−1^	Yield/%
0.10	11.0 ± 1.0	100
0.20	14.0 ± 1.0	70
0.30	21.0 ± 1.6	70
0.40	30.0 ± 2.1	75
0.50	31.0 ± 1.6	62
0.56	36.0 ± 2.4	64

**Table 2:** Properties and composition of PMMA/DODAB/PDDA NPs: mean hydrodynamic diameter (Dz), zeta-potential (*ζ*), polydispersity (P), and conductance (G) after dialysis or after removal of free cationic components. Subscript P indicates concentration in NPs; asterisk indicates data for non-treated NPs after dialysis. Concentrations are given in mg mL^−1^.

Dz/nm	*ζ*/mV	P	G/µS	[PMMA]_P_	[DODAB]_P_	[PDDA]_P_
225 ± 2 *	70 ± 1*	0.10 ± 0.02 *	92 ± 3 *			
217 ± 1	73 ± 1	0.12 ± 0.01	4 ± 2	7.0 ± 0.7	0.32 ± 0.03	0.70 ± 0.02

**Table 3:** Comparison of IgG1 and IgG2a production and footpad swelling (FS) induced by the delayed type hypersensitivity reaction (DTH) to OVA alone or carried by three different cationic nanoparticles NPs: DODAB bilayer fragments (BF) [44], PDDA/OVA NPs [20], and PMMA/DODAB/PDDA NPs (this work). IgG1 and IgG2a production were depicted from absorbance at 492 nm (A492nm) determined at the serum dilutions (in brackets) and days 14 and 21 after immunization.

Assembly	Time/Days	A_492nm_ (IgG1)	A_492nm_ (IgG2a)	FS/mm
OVA	14	0.40 (1/128)	0.10(1/32)	0.3
	21	0.25 (1/128)	0.10(1/32)	
DODAB BF/OVA	14	1.45 (1/128)	0.70(1/32)	1.3
	21	0.80 (1/128)	0.40(1/32)	
PDDA/OVA	14	1.20 (1/256)	0.15(1/8)	0.5
	21	1.50 (1/256)	0.50(1/8)	
NPs/OVA	14	1.90 (1/512)	0.60(1/8)	0.9
	21	1.90 (1/512)	0.50(1/8)	

## Data Availability

All data available are reported in the article.
